# A Case of Bone Marrow Transplant-Associated Partial Lipodystrophy

**DOI:** 10.7759/cureus.71641

**Published:** 2024-10-16

**Authors:** Zakiyyah Khan, Kabeer Ali, Aliyyah Khan, Surujpal Teelucksingh

**Affiliations:** 1 Internal Medicine, Medical Associates Hospital, Saint Joseph, TTO; 2 Internal Medicine, Eric Williams Medical Sciences Complex, Champs Fleurs, TTO; 3 Anesthesiology, Eric Williams Medical Sciences Complex, Champs Fleurs, TTO; 4 Faculty of Clinical Medical Sciences, The University of the West Indies, Saint Augustine, TTO

**Keywords:** acquired lipodystrophy, bone marrow transplant (bmt), lipodystrophy, partial lipodystrophy, post bone marrow transplant complications

## Abstract

Lipodystrophy is characterized by abnormal fat distribution and has a broad range of etiologic associations. We present a case of a young Afro-Caribbean female to highlight the clinical features of bone marrow transplant-associated partial lipodystrophy. This review examines and provides diagnostic recommendations for discerning lipodystrophy and its potential cause and also provides follow-up guidance in those diagnosed with bone marrow transplant-associated partial lipodystrophy. We utilize this case to highlight the clinical implications of lipodystrophy and create awareness of this as a potential outcome in childhood cancer survivors.

## Introduction

Lipodystrophy (LD) is accepted as a rare, heterogeneous condition defined by aberrant fat distribution, which can be generalized, partial, or localized. It has many etiologic associations, including congenital syndromes, autoimmune and inflammatory conditions, or drug-induced disease [1]. LD is often subclassified by these associations and by the extent of fat redistribution throughout the body. Few reports of lipodystrophy are attributed to bone marrow transplantation (BMT). Given the wide variability of these conditions and the limited literary accounts of such presentations, it can be difficult to ascertain the most likely cause of lipodystrophy in presenting patients.

Diagnosing lipodystrophy is crucial because the condition significantly increases the risk of severe metabolic complications. Lipodystrophy leads to abnormal fat distribution, impairing the body’s ability to regulate insulin and fat storage. This imbalance often results in insulin resistance, diabetes, hypertriglyceridemia, and fatty liver disease [1], all of which raise the risk of cardiovascular problems. Early diagnosis allows for timely management of these metabolic issues, improving overall health outcomes and reducing the risk of long-term complications.

The following case report looks at one such uncommon presentation of partial lipodystrophy in a young female with a history of bone marrow transplantation, and based on a literature review, we have suggested an algorithm to aid in the diagnosis of bone marrow transplant-associated partial lipodystrophy. We also suggest greater recognition of this specific form of lipodystrophy as a complication of bone marrow transplant in childhood cancer survivors.

## Case presentation

A 36-year-old female presented with a two-year history of progressive weight redistribution, defined by weight loss to the gluteal region and lower limbs as well as weight gain to the face, trunk, and upper limbs.

She has a history of stage II A nodular sclerosis Hodgkin lymphoma and a negative history of autoimmune, metabolic, or infectious diseases. The diagnosis of Hodgkin lymphoma was made 18 years prior to presentation, after complaints of right neck swelling and weight loss. She was exposed to multiple rounds of chemotherapy, initially with Adriamycin/doxorubicin, bleomycin, vinblastine, and dacarbazine (ABVD) regimen, after which her malignancy persisted. She received cyclophosphamide, Oncovin/vincristine, procarbazine, and prednisone (COPP) protocol chemotherapy that led to the complete remission of the disease.

Seven years later, there was a recurrence of her lymphoma with bone marrow involvement. She received etoposide, methylprednisolone/solumedrol, high-dose cytarabine/ara-C, and cisplatin (ESHAP) chemotherapy regimen again achieving complete remission, followed by total body irradiation and bone marrow transplantation. She subsequently developed primary hypothyroidism and was started on thyroid hormone replacement.

**Figure 1:**
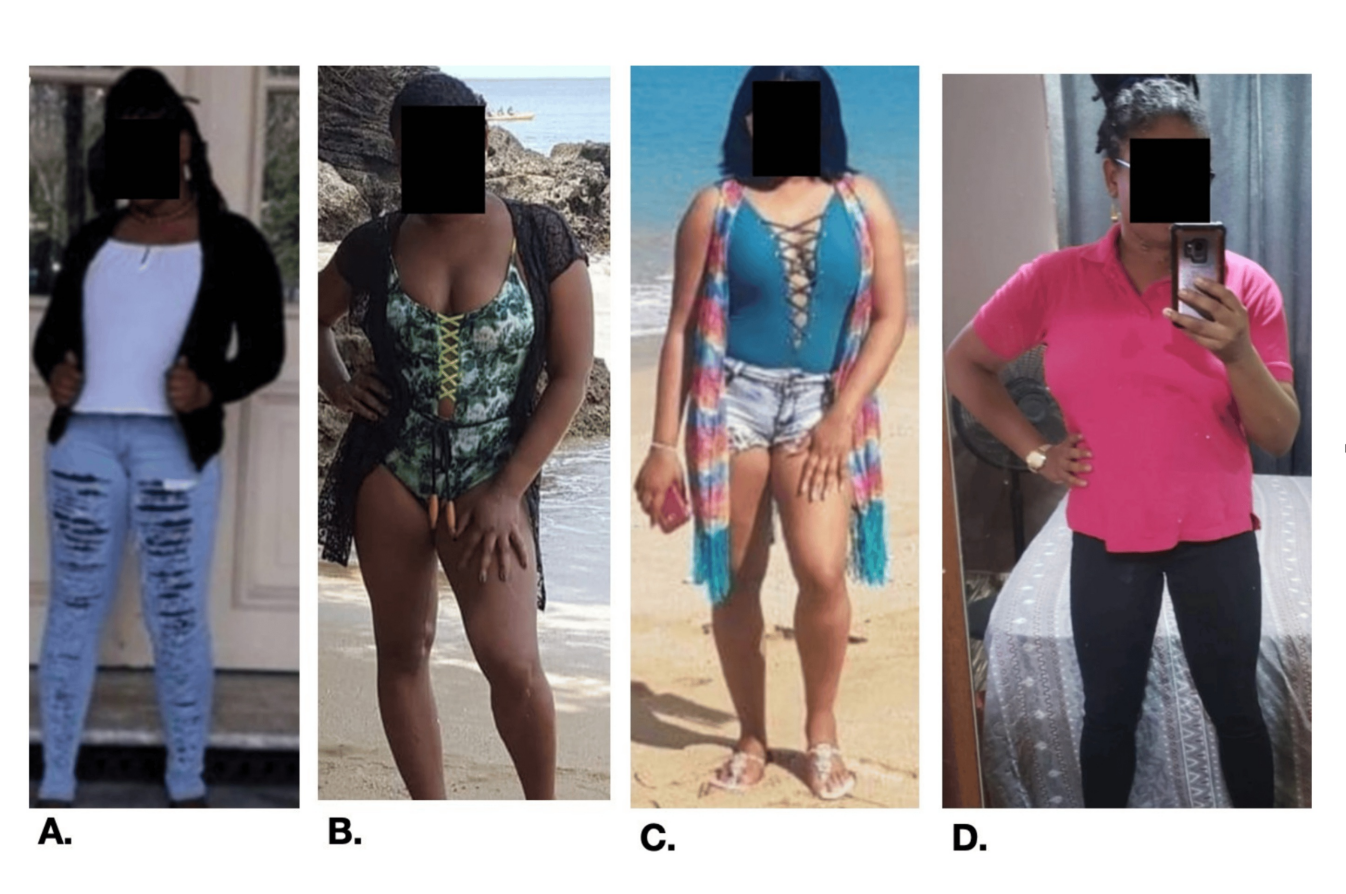
Weight redistribution in patient over two years. (A) Patient’s phenotypic appearance prior to bone marrow transplant. (B) and (C) Phenotypic appearance one year after bone marrow transplant; patient noted weight gain in the upper body. (D) Phenotypic appearance two years following bone marrow transplant; patient noted weight loss in the lower body.

Over two years, she experienced progressive symmetric weight loss in her lower limbs and persistent weight gain in her upper body (Figure 1) despite rigorous exercise, dietary changes, and medication compliance. She had no significant associated symptoms.

**Figure 2:**
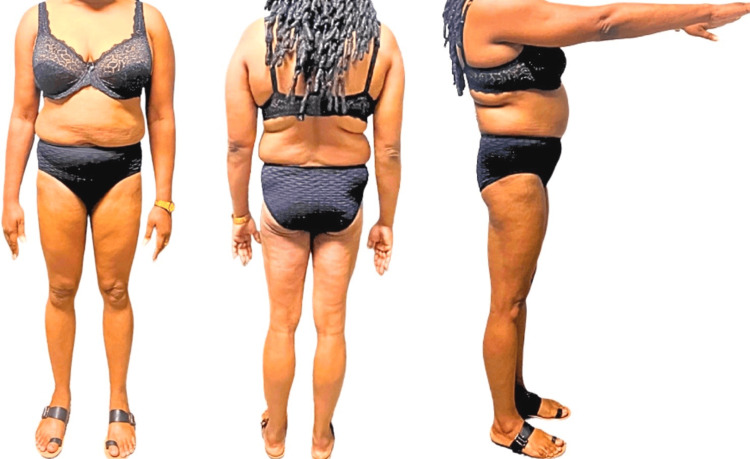
Anterior, posterior, and right lateral views of the phenotypic appearance of a 36-year-old Afro-Caribbean female with significant lipoatrophy to the gluteal region and lower limbs and excessive fat distribution to the trunk and upper arms.

On examination, the patient had symmetric lower extremity lipoatrophy, most marked in the gluteal region and thighs. Lipohypertrophy was seen in the upper extremity, particularly in the trunk and arms (Figure 2).

She had a PET-CT scan done, which showed no evidence of lymphoma recurrence or other significant abnormalities. She had a dual-energy X-ray absorptiometry (DEXA) scan Z-score of -0.6. No other imaging was done. Her low-density lipoprotein (LDL) cholesterol is mildly elevated and persists despite lifestyle modification. Other lipid parameters, cortisol, and comprehensive metabolic panel are normal. HbA1c is 5.4%. Treatment was started on a statin. 

## Discussion

Lipodystrophy (LD), by definition, is the abnormal distribution of fat. The associated conditions are rare, heterogeneous diseases characterized by this aberrant fat redistribution. Current data suggest that there are no definitive diagnostic criteria for lipodystrophy; its pathophysiology is poorly understood and treatment is limited in efficacy and focused on prevention and management of the significant co-morbid complications [1].

Few research reports have attempted to formulate diagnostic approaches to lipodystrophy. Their methods heavily rely on clinical features for diagnosis and determining the associations with metabolic conditions. One consistency seen in the literature is that LD can be classified as congenital or acquired causes with generalized, partial, or localized patterns of fat aberrancy. This classification is useful when the associated etiology of the disease is unclear.

Each sub-classification has its own set of patterns of presenting features (Table 1): Congenital generalized lipodystrophy (CGLD) syndromes are associated with genetic mutations [2], and they have a typical appearance of generalized near absence of fat seen in the first two years of life. Congenital partial lipodystrophy (CPLD) or familial partial lipodystrophy (FPLD) has patterns of simultaneous fat hypertrophy to the upper body and atrophy in the extremities. There are several subtypes based on the distribution of aberrancy, age of onset, associated genetic mutations, and associated complications [3]. Acquired lipodystrophic syndromes have generalized, partial, or localized patterns of fat aberrancy secondary to another condition, albeit autoimmune, infectious [4], or with drug use [5].

**Table 1:** Sub-classification of congenital and acquired lipodystrophy. AGPAT2: 1-acylglycerol-3-phosphate-O-acyltranserfase 2; BSCL2: Berardinelli-Seip congenital lipodystrophy type 2; CAV1: caveolin 1; PTRF: polymerase I and transcript release factor; FPLD: familial partial lipodystrophy; PPARG: peroxisome proliferate-activated receptor gamma; PLIN1: perilipin 1; CIDEC: cell death-inducing DFFA-like effector c; LIPE: lipase E, hormone-sensitive type.

Category	Distribution	Subtypes
Congenital [2,6]	Generalized	Genetic mutations: AGPAT2, BSCL2, CAV1, PTRF
	Partial	FPLD 1: Kobberling type
		FPLD 2: Dunnigan type
		FPLD 3: PPARG gene
		FPLD 4: PLIN1 gene
		FPLD 5: CIDEC gene
		FPLD 6: LIPE gene
Acquired [7]	Generalized	Panniculitis, autoimmune, idiopathic
	Partial	Barraquer-Simons syndrome, HIV-associated drug-induced
	Localized	Panniculitis, drug injections, idiopathic

The patient experienced a young-adult onset of fat redistribution, exhibiting significant lipoatrophy in both the gluteal region and the lower limbs, both symmetrically. In addition, there was lipohypertrophy to the upper chest, back, neck, face, and arms bilaterally. From the described classification above, her physical findings were similar to FPLD, particularly Dunnigan subtype 2.

Araújo-Vilar and Santini [1] adapted and expanded on this simple classification to create a comprehensive algorithm for diagnosing the subtypes of LD. Their research included an unusual association of lipodystrophy, bone marrow transplant-associated lipodystrophy (BMTAL) seen with similar phenotypic features to FPLD type 2, in which both conditions have the same distribution of fat aberrancy; but with BMTAL, there is the prior patient history of total body irradiation, chemotherapy, and bone marrow transplant exposure. This addition of BMTAL was based on Santini’s [7] own research on patients developing fat redistribution following bone marrow transplantation (BMT) as well as another study by Adachi that demonstrated partial LD resembling Dunnigan type FPLD following bone marrow transplantation [8].

In 2021, Tews et al. [9] outlined and summarized seven case reports, including the above-mentioned cases of lipodystrophy, as a late effect of bone marrow transplantation. These reports demonstrated many similarities to our case as they all had childhood or young-adult onset of primary malignancy that required BMT and subsequently developed partial lipodystrophy with fat loss in the extremities and lipohypertrophy in the face, neck, and abdomen. These patients received intensive chemotherapy and total body irradiation in addition to BMT. Consequentially, all cases in this study experienced thyroid and/or gonadal deficiencies, requiring replacement therapy.

There were, however, a few significant differences in this study compared to our patient. Patients had primary diagnoses of acute lymphoblastic leukemia, acute myeloblastic leukemia, or neuroblastoma compared to our patients who had Hodgkin lymphoma. Our patient had a later age of onset of LD compared to the cases in this study, but also a shorter gap between BMT and LD onset, approximately two years compared to the gap variation of 10 to 28 years later in their studies. Our patient is also the only Afro-Caribbean female to our knowledge reported to date.

Survivors of childhood cancers and those treated with chemotherapy, total body irradiation, and bone marrow transplantation have been shown to have a substantially high burden of many late adverse effects and chronic complications affecting overall quality of life. It is standard that this group of patients be monitored routinely in their lifetime for such potential complications based on their primary disease and forms of treatment exposure. Literature on such surveillance fails to acknowledge lipodystrophy as a complication of BMT or recognize its likelihood to increase the risk of metabolic disease in these patients [10,11].

The initial recommendation is that there should be inclusion of this phenomenon as a potential late outcome among survivors of total body irradiation conditioning and subsequent BMT [12,13]. Adachi et al. [8] proposed that the combination of chemotherapy, total body irradiation, and BMT factors might have led to the disease by destruction of adipocyte function in the subcutaneous fat limiting storage capacity and leading to the development of lipodystrophy; however, studies are not conclusive.

**Figure 3:**
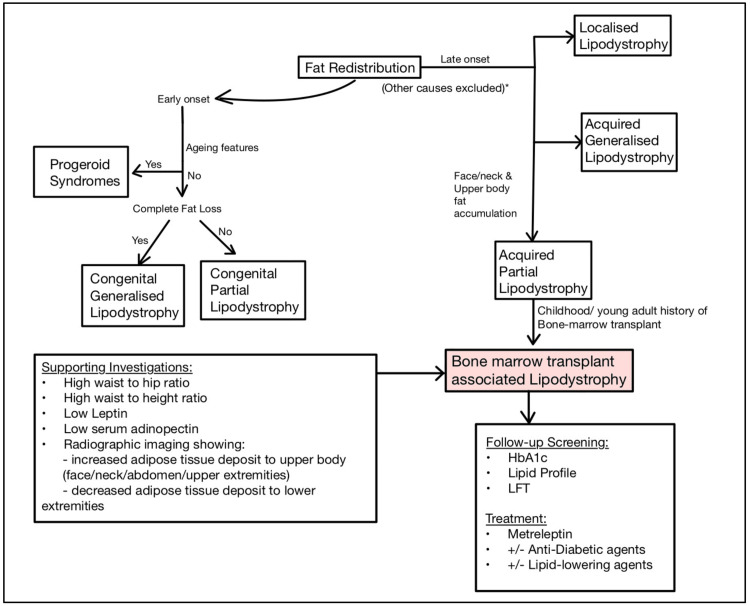
A simplified algorithm to diagnosing bone marrow transplant-associated lipodystrophy. In a patient with a history of fat redistribution and bone marrow transplantation, bone marrow transplant-associated partial lipodystrophy must be considered. Supporting investigations can include serial anthropometric measurements, low leptin, low serum adiponectin, and radiographic imaging. Routine screening and treatment of metabolic disease sequelae is paramount.

In BMT risk-associated patients, screening for lipodystrophy may be clinically based, with objective anthropometric assessment, particularly of the abdomen and extremities, for increasing waist-to-hip and waist-to-height ratios [14]. DEXA scan, fully body MRI, or CT imaging may be done as further supporting investigations highlighting aberrant fat redistribution [14]. Screening for disease development may also be done by trending for a decline in adipokines such as leptin and adiponectin [15]. The frequency of follow-up screening should be on an individual basis, guided by surveillance recommendations for primary disease and its treatment type as well as objective individual clinical judgment (Figure 3).

Once a diagnosis of bone marrow transplantation-related lipodystrophy has been established, by exclusion of other causes of fat redistribution and the aforementioned supporting investigations, patients should be screened routinely for metabolic complications of this disease such as diabetes mellitus, dyslipidemia, fatty liver disease, and cardiovascular dysfunction [16,17]. Guidelines should be established on the frequency of screening in this subset of patients.

Treatments for LD aim to ameliorate the metabolic disturbance and the pathological changes with fat redistribution. Lifestyle modifications are crucial to the slow progression of the development of metabolic complications, and pharmacologic interventions should be introduced accordingly. Dietary recommendations include following a balanced macronutrient composition with limited fat intake. Intense exercise should be encouraged if there are no contraindications. There should be a low threshold for commencing anti-diabetic and anti-hyperlipidemic medications in these patients. Routine follow-up is encouraged for monitoring such high morbidity long-term complications [18].

Metreleptin, a recombinant analog of the human hormone leptin, has proven beneficial in the treatment of LD [19,20]. It leads to favorable changes in body composition, improved insulin sensitivity, hypertriglyceridemia, and transaminitis, reversing and limiting metabolic complications in patients refractory to standard medications for treating metabolic diseases. However, drug availability and high costs limit its application for treatment. 

## Conclusions

There are significant gaps in understanding, diagnosing, and treating lipodystrophy, and there is no definitive method to associate this patient’s presentation to bone marrow transplant-associated lipodystrophy. However, it is the least confounding explanation for the findings seen in our patient; thus, it is fair to be guided in continuing this patient’s care to limit the long-term complications that can be associated with lipodystrophy. She is followed every six months with repeat lipid profiles, liver function tests, and HbA1c.

It is imperative to highlight the recurring theme of metabolic complications associated with LD. Insulin resistance, diabetes mellitus, hypertriglyceridemia, low HDL, and hepatic steatosis are prevalent in many subtypes of lipodystrophy. Hence, continued follow-up is necessary in this subset of patients to identify these associations and treat them accordingly to limit morbidity.
